# The Current State of Subjective Training Load Monitoring: Follow-Up and Future Directions

**DOI:** 10.1186/s40798-022-00433-y

**Published:** 2022-04-15

**Authors:** Joseph O. C. Coyne, Aaron J. Coutts, Robert U. Newton, G. Gregory Haff

**Affiliations:** 1 School of Medical and Health Sciences, Edith Cowan University, Joondalup, WA 6027 Australia; 2 Human Performance Research Centre, University of Technology Sydney (UTS), Moore Park Rd, Moore Park, NSW 2021 Australia; 3 School of Sport, Exercise and Rehabilitation, University of Technology Sydney (UTS), Moore Park Rd, Moore Park, NSW 2021 Australia; 4 Directorate of Psychology and Sport, University of Salford, Salford, Greater Manchester UK; 5 18 Bondi Pl, Kingscliff, NSW 2487 Australia

**Keywords:** Training load, Perceived exertion, Sport performance

## Abstract

This article addresses several key issues that have been raised related to subjective training load (TL) monitoring. These key issues include how TL is calculated if subjective TL can be used to model sports performance and where subjective TL monitoring fits into an overall decision-making framework for practitioners. Regarding how TL is calculated, there is conjecture over the most appropriate (1) acute and chronic period lengths, (2) smoothing methods for TL data and (3) change in TL measures (e.g., training stress balance (TSB), differential load, acute-to-chronic workload ratio). Variable selection procedures with measures of model-fit, like the Akaike Information Criterion, are suggested as a potential answer to these calculation issues with examples provided using datasets from two different groups of elite athletes prior to and during competition at the 2016 Olympic Games. Regarding using subjective TL to model sports performance, further examples using linear mixed models and the previously mentioned datasets are provided to illustrate possible practical interpretations of model results for coaches (e.g., ensuring TSB increases during a taper for improved performance). An overall decision-making framework for determining training interventions is also provided with context given to where subjective TL measures may fit within this framework and the determination if subjective measures are needed with TL monitoring for different sporting situations. Lastly, relevant practical recommendations (e.g., using validated scales and training coaches and athletes in their use) are provided to ensure subjective TL monitoring is used as effectively as possible along with recommendations for future research.

## Key Points

- Subjective measures of training load are recommended to be included in bespoke decision-making frameworks for different sporting contexts and to complement coaching decisions due to their efficacy in measuring psychophysiological responses to training, low cost, and ease of use.
- When calculating subjective training load, exponentially weighted moving averages may not have any greater relationship with performance compared to simple moving averages but may be more useful as they can be calculated much sooner.
- To compare “apples with apples” as best as currently possible with internal and external training load, it is recommended to use the training impulse (the product of training volume and intensity factors) for both internal and external load, rather than a singular volume or intensity factor.
- Modeling approaches that account for the magnitude of outcome measures, rather than just binomial outcomes, and the lagged effect of multiple concurrent time series (e.g., training load and performance) on one another should be considered.
- Any subjective measurement method should be validated, applied as intended (e.g., using verbal anchors to obtain a numerical rating) and combined with education tools like Borg’s blackness test to obtain the best results for athletes and coaches.

## Introduction

Training load (TL) monitoring is normally applied to assess the physical work an athlete performs in training (i.e., external load) and the athlete’s within-training response to that physical work (i.e., internal load) [1, 2]. Sessional ratings of perceived exertion (sRPE) and differential ratings of perceived exertion (dRPE) are both subjective measures of the intensity of internal TL [1, 3]. Sessional ratings of perceived exertion, which are seen as a global measure of perceived exercise intensity [4, 5], seem to be the most used measure in practice; being often recommended as the primary TL measure in team sports and being widely employed in endurance sports [6–8]. Meanwhile, it is proposed that dRPE distinguishes perceptual responses according to specific local or central mediators in training or games (e.g., leg exertion, breathlessness exertion, technical exertion) [9]. While dRPE may be more sensitive to different facets of internal load, it is unknown whether dRPE can be used to provide a global intensity measure (similar to sRPE), although a combination of dRPE scores seem to explain the majority of the variance in sRPE scores (76% and 66–91%) in two different studies [9, 10]. Besides sRPE and dRPE, there are other methods of subjectively evaluating an athlete’s response to training (e.g., athlete self-report measures of perceived wellness/stress [11], an experienced coach’s observations of how an athlete has performed in training); however, these are not considered TL measures nor normally used in TL models. This is primarily due to these measures not being a direct quantification of TL. These other subjective measures also appear to be more often applied as assessments of an athlete’s readiness to train or perform.

In our previous publication “*The Current State of Subjective Training Load Monitoring — a Practical Perspective and Call to Action*” [12], there were a number of key issues that we suggested should be considered when implementing a subjective TL monitoring program. These key issues can generally be categorized into three themes: (1) calculations of TL (“*does it matter how we calculate TL?*”), (2) performance relationships (“*can we model sports performance from subjective TL?*”)*,* and (3) types of decision-making tools for practitioners (“*where does subjective TL monitoring fit in an overall decision-making framework?*”)*.* Since our paper’s publication [12], there have been a number of investigations in these areas that have added to the current body of scientific literature, but these key issues remain important to consider for both practitioners and researchers*.* As such, the purpose of this article is to further discuss these issues and provide pragmatic strategies for effectively using subjective TL monitoring techniques. For a comprehensive background to the information presented from this point in this article, we suggest readers review our previous publication “*The Current State of Subjective Training Load Monitoring—a Practical Perspective and Call to Action*” [12].

## Does it Matter How We Calculate Training Load?

There are several different considerations practitioners and researchers should be aware of when calculating TL variables. The first is the arbitrary lengths of longer-term chronic positive “fitness” and shorter-term acute negative “fatigue” periods [13, 14]. Based on research examining different acute and chronic timeframes and their relationship with injury risk [15, 16], we have previously suggested practitioners can adjust the length of acute and chronic periods to the length of their preferred training micro- and meso-cycles. Although this is a simplistic solution, our recent research using acute and chronic periods based on micro- and meso-cycle length demonstrated significant moderate to large correlations between TL and performance and differences in TL of higher and lower performers in track and field [17], weightlifting [18] and basketball [19]. Despite these results, it is still worth considering individualizing the acute and chronic periods to different sports and different athletes within the same sport. It would seem worthwhile to identify the best-fitting period lengths for individual athletes or teams modeled against competitive performance using impulse-response models [14, 20] or variable selection procedures with measures of model-fit like the Akaike Information Criterion (AICc) [21], which Tysoe et al. [22] employed recently in their paper examining bowling training loads and injury risk. By identifying the best-fitting acute and chronic period lengths for competitive performance, this may help practitioners better adjust any planned increases or decreases to an athlete’s TL and may also provide potential feedback on optimal microcycle, mesocycle and taper lengths to sports coaches. It should be realized that the functional status of an athlete (e.g., recent training and injury history, current nutritional practices) will vary from period to period and an athlete’s optimal acute and chronic period lengths for performance may be dynamic in nature and require re-examination at specific intervals (e.g., the start of each training year).

The next issue with calculating TL variables is deciding which smoothing method to use. Currently, there are three main smoothing methods that have been presented in the scientific literature: a simple moving or “rolling” average (SMA) [13] and two different exponentially weighted moving averages (EWMA), as per Williams et al. (EWMA-W) [23] and as per Lazarus et al. (EWMA-L) [24]. These different smoothing methods all produce different TL values for the acute and chronic time periods. The key difference between EWMA and SMA is EWMA gives an increased weighting to the most recent TL completed by the athlete in any given period whereas there is an even weighting of the TL over that same period when the SMA is employed. Meanwhile, the key difference between the two EWMA methods is that EWMA-L gives a weighted average that has a higher correlation with SMA [24] and will give more weight to the less recent TL in the acute or chronic time period than EWMA-W. Despite each smoothing method having conceptual issues [12], the EWMA methods have generally been suggested as a more suitable smoothing approach, mainly due to EWMA more accurately representing the decaying physiological nature of fitness and fatigue in calculations, when compared to a SMA [23, 25]. Additionally, interpretation of existing research suggests that EWMA TL variables have a greater relationship to injury risk [22, 25] when compared to SMA TL variables; however, a better relationship to performance outcomes in our recent research with EWMA compared to SMA has been mixed [18, 19]. Comparable to determining the lengths of the acute and chronic periods, we recommend variable selection procedures using model-fit measures like AICc against performance or injury outcomes to determine the optimal smoothing method for the sport and/or athlete. Evaluating optimal smoothing methods may also be worthwhile at specific intervals, like acute and chronic period lengths.

When using variable selection procedures based on model-fit, practitioners and researchers should consider if different acute and chronic period lengths and smoothing methods are appropriate for the sport and athlete(s) they are working with. For instance, EWMA can be calculated in practice much sooner than SMA, especially if dealing with longer chronic periods; and this becomes an important consideration for sports that have short or intermittent preparation periods before competitions. One such example would be fight-camps in mixed martial arts, where historically athletes may enter the fight-camp having done little training prior and the fight-camp then typically only lasting ~ 6–8 weeks. In this case, using a SMA to calculate TL may be of little use, considering acute and chronic TL may not be able to be compared until most of the fight-camp is complete.

After the period length and method of smoothing are determined, practitioners and researchers should consider which change in TL measure to apply. Although it may be reasonable to use the acute-to-chronic workload ratio (ACWR) as a very general approximation of any changes in TL, its use in any other setting is highly questionable [26]. As such, other measures such as training stress balance (TSB) [27] and differential load [24] are preferred over the ACWR. Training stress balance is calculated as the difference between the chronic and acute periods, is similar to Banister’s original training impulse model [14] and seems to have served as the genesis for the ACWR [28]. Meanwhile, the differential load is an exponential smoothing of week-to-week rate of change in TL [24]. Like the most appropriate smoothing methods and acute/chronic period lengths, we suggest using model-fit measures like AICc against a variable of interest (i.e., performance) to determine the change in TL method best suited for the sport and/or athlete.

To provide an example of a variable selection process, we have used two previously published datasets from elite international Olympic athletes (long jump [17], and basketball [19]) investigating repeated measure performance outcomes (World Athletics performance scores for long jump and coach ratings for basketball) prior to and during the 2016 Olympic Games with the use of a sRPE-TL (sRPE * training duration). More details on the datasets are provided in Coyne et al. [19] and Coyne et al. [17] along with their approval for use by the Edith Cowan University Human Ethics Committee (Approval #19521). The variable selection process was accomplished using the *AICcmodavg* package in *R* (version 3.6.3, R Foundation for Statistical Computing, Vienna, Austria) and was designed to find the TL measures with the lowest AICc when modeled as an explanatory variable for performance outcomes. The AICc compares models for goodness of fit while also accounting for the simplicity of the model and a lower AICc suggests a higher quality model, relative to other models assessed [21]. In this process, performance outcomes were exponentiated in the models to allow for the saturation effect of training on performance [20]. Further, as the datasets contained repeated measures of performance, a mixed effect model with the athlete as the random intercept was used. Alongside this in the variable selection process, all TL measures were smoothed using SMA, EWMA-W and EWMA-L, acute period lengths ranged from 5 to 9 days (in 2-day increments), chronic period lengths 14–42 days (in 7-day increments) and taper lengths 7–28 days (again in 7-day increments) [29, 30]. The TL measures were divided into the following conceptual categories: (1) acute TL, (2) chronic TL, (3) change in TL (TSB or differential load) and (4) taper length. The results of the variable selection process are presented in Table 1.

**Table 1:** Akaike Information Criteria [21] variable selection for different categories of training load explanatory variables modeling performance outcomes in two different groups of elite international athletes

Order	Acute TL	AICc	Chronic TL	AICc	Change in TL	AICc	Taper	AICc
Athletics (*n* = 4, performance measures = 29)								
1	5-d EWMA-W	124	42-d EWMA-L	123	TSB9:14-d EWMA-L	120	TSB9:14-d SMA CH28-d	113
2	5-d SMA	124	35-d EWMA-L	123	TSB7:14-d EWMA-L	121	TSB7:14-d SMA CH28-d	113
3	7-d EWMA-W	125	28-d EWMA-L	123	TSB9:14-d EWMA-W	122	TSB9:14-d EWMA-W CH28-d	113
4	9-d EWMA-W	125	42-d EWMA-W	124	TSB9:21-d EWMA-L	122	TSB9:14-d EWMA-L CH28-d	114
5	5-d EWMA-L	125	21-d EWMA-L	124	TSB7:21-d EWMA-L	122	TSB7:14-d EWMA-W CH28-d	114
6	7-d EWMA-L	125	35-d EWMA-W	124	TSB7:14-d EWMA-W	122	TSB9:42-d EWMA-L CH28-d	114
7	9-d EWMA-L	125	42-d SMA	125	TSB9:21-d EWMA-W	122	TSB9:21-d SMA CH28-d	114
8	7-d SMA	125	28-d EWMA-W	125	TSB9:35-d EWMA-W	123	TSB9:35-d EWMA-L CH28-d	114
Basketball (*n* = 13, performance measures = 171)								
1	9-d SMA	541	28-d SMA	538	TSB9:28-d SMA	527	TSB5:21-d SMA CH21-d	498
2	7-d EWMA-W	543	14-d SMA	542	TSB7:28-d SMA	532	TSB9:21-d SMA CH21-d	500
3	9-d EWMA-W	543	35-d SMA	542	TSB9:21-d SMA	533	TSB7:21-d SMA CH21-d	501
4	5-d EWMA-L	543	14-d EWMA-W	544	TSB9:35-d SMA	534	TSB9:14-d EWMA-W CH21-d	504
5	5-d EWMA-W	543	35-d EWMA-L	545	TSB9:14-d EWMA-L	534	TSB5:28-d SMA CH21-d	505
6	7-d EWMA-L	544	21-d SMA	545	TSB7:14-d EWMA-L	535	TSB7:14-d EWMA-W CH21-d	507
7	9-d EWMA-L	545	28-d EWMA-L	545	TSB9:21-d EWMA-W	536	TSB9:21-d EWMA-W CH21-d	507
8	7-d SMA	545	42-d EWMA-L	545	TSB5:14-d EWMA-L	536	TSB7:21-d EWMA-W CH21-d	508

*TL* training load, *AICc* Akaike Information Criterion, *TSB* training stress balance, *CH* change in TL measure prior to competition, *d* days, *SMA* simple moving averages, *EWMA-W* exponentially weighted moving average as per Williams et al. [23], *EWMA-L* exponentially weighted moving average as per Lazarus et al. [24]

From the results of a variable selection process, the best combination of acute, chronic, and taper period lengths across the categories can be identified. We suggest that these combinations should correspond to one another. For example, a 21-day chronic TL would not be used with a 9:42-day TSB, nor a 21-day chronic TL SMA used with a 9:21-day TSB EWMA-W. For these datasets (Table 1), it seems the best combinations of period length and smoothing method based on the lowest AICc values are as follows: (1) athletics: 9-day acute, 14-day chronic, 28-day taper period (SMA), and (2) basketball: 9-day acute, 21-day chronic, 21-day taper period (SMA). Of note from these datasets is that the overall combination of SMA TL variables had superior fit (i.e., lower AICc) to performance in the datasets compared to EWMA-W and EWMA-L. It is worthwhile mentioning that these time periods and smoothing methods are only examples based on the datasets described above. We believe that practitioners and researchers should not solely rely on the information derived from the above models (even if working in the same sports) but use similar methods to establish their own optimal time periods and smoothing methods with the sports they work with. It is also worthwhile considering using similar methods for individual athletes; especially if working within an individual sport with small training squad numbers and there are sufficient performance outcomes to generate a sound model. Any results gleaned from these methods should be viewed with established principles of training periodization in mind and, perhaps most importantly, ensure that their application remain practical for sporting coaches to implement in training programs.

Another suggested practice in TL monitoring is examining the relationship between internal and external TL to optimize an athlete’s training [1, 12]. Theoretically, a consistent trend of greater external TL with similar or lower internal TL responses over time would represent a positive adaptation to the training process [31]. Meanwhile a trend of increasing internal TL compared to a similar or lower levels of external TL may indicate a negative training adaptation [31]. This suggestion is reinforced by the results of our previous research in basketball [19] with the training efficiency index (TE_*I*_) [31], which quantifies the relationship between external and internal training load, having the largest correlations with athlete in-game performance, when compared to internal (subjective) and external TL measures alone.

However, when examining this internal–external TL relationship, we suggest caution when choosing which measures are used. For instance, it is common for internal TL to be calculated as a training impulse in research (i.e., the product of an intensity factor [e.g., sRPE] and a volume/duration measure [e.g., kilometres or total time]) [5]. However, it seems uncommon for external TL to be quantified as a training impulse with either intensity or volume/duration measures being most used (e.g., amount of balls thrown, total distance, total high speed running distance, total PlayerLoad™, PlayerLoad™ per minute) [32]. This may be an issue for any studies examining internal–external TL relationships if in the study, the external TL is not computed as a training impulse like internal TL. We suggest practitioners and researchers examining this relationship may need to ensure external “apples” are being compared with internal “apples”; with the “apples” in this case being the product of training volume/duration and intensity. Further, considering the genesis for modern TL monitoring systems from Banister’s training impulse model [14], there may also be issues (e.g., lack of sensitivity) when examining external measures and the relationship to an outcome of interest, if those external measures are a singular intensity or volume/duration measure. Considering the multitude of factors that affect athlete training adaptations [12] and that even TL, regardless of whether it is internal or external, as a product of intensity and duration is a relatively simplistic and somewhat limited tool for accurately modeling training responses in elite athletes [33], this may be one of the many reasons for the inconsistent results [26] in research examining the relationship between TL and injury or performance.

## Can We Model Sports Performance from Subjective Training Load?

Although adequately defining performance can be difficult, especially in open skill team sports [34], we advocate that practitioners and researchers should aim to examine any relationship between subjective TL and performance with performance measures from the athlete’s actual competition, in preference to physical tests (e.g., a countermovement jump) or other markers of athlete readiness (e.g., heart rate variability). While both have been used as surrogates for actual competitive performance, physical tests and athlete readiness markers may be unrelated to performance in some scenarios (e.g., heart rate variability can be negatively related to performance depending on the level of athlete) [34, 35]. When considering competition performance measures, the level of evidence for a relationship between subjective TL and performance seems to have been strengthened by several recent publications [18, 19]. It should be mentioned that this evidence is based on correlations between performance and TL or differences in TL between successful and unsuccessful performances in case/observational studies [17–19]. Beyond correlations or differences in means, using mixed models that contain repeated measures of performance (from the same athletes) to identify if TL measures are significant explanatory variables for performance in more controlled studies would seem to be a possible next step to further examine the relationship between subjective TL and performance.

To provide practitioners and researchers with potential examples of how these mixed models may be created, we have created two models using the same previously mentioned datasets (long jump [17] and basketball [19]). Both models were created using the *lmerTest* package in *R* and performance outcomes were exponentiated to allow for the saturation effect of training on performance [20]. These models both contained the athlete as a random intercept. The explanatory variables were chosen from the same variable selection process described above (i.e., the combination of variables with the lowest AICc) and were divided into the same conceptual categories. For multi-collinearity reasons, consideration was also given to how the TL variables in the models related to one another. For example, as TSB is the difference between acute and chronic TL, we decided to include only chronic TL, rather than both acute and chronic TL, in the models for parsimony reasons. The percentage of training burdened by injury or illness in the last 21-days was also included as an explanatory variable, as this was available across both datasets and would help quantify the influence of TL on performance outcomes independent of injury and illness burden [18, 19].

The model summaries for each of the datasets are presented in Table 2. All models were checked for (a) linearity, (b) residual independence, (c) residual normality and (d) multicollinearity with a variance inflation factor below 4 being deemed adequate [36]. Effect sizes of the variables were determined using marginal *f*^2^ [37] and interpreted as trivial (< 0.02), small (0.02–0.14), moderate (0.15–0.34) and large (> 0.35) [38]. Again, we caution that due to the size of these two datasets, the models in these examples have been used only for explanatory purposes and the results are only specific to these groups of athletes. However, the use of training and test datasets or cross-validation of models is recommended as datasets grow large enough to do so. Other limitations to these models are that they do not consider the type of taper used (e.g., step, exponential) [29], they only consider internal TL and there is only a consideration of the percentage of total training burdened by injury without any respect to the location or severity of the injury/illness that caused this burden. In particular, the inclusion of external training load and its relationship with internal load (e.g., TE_*I*_) [31] in models examining TL-performance relationships is suggested; if those data are available.

**Table 2:** Model summaries for performance outcomes in two different groups of elite international athletes

Variable	Estimate [95% CI]	Standard error	Pr( >|*t*|)	*f*^2^	Effect size
Athletics (*n* = 4, performance measures = 29)					
(Intercept)	0.724 [− 0.872, 2.321]	0.815	0.38		
Chronic 14-d SMA	0.002 [− 0.003, 0.008]	0.003	0.49	0.008	Trivial
TSB 9:14-d SMA	0.019 [0.003, 0.035]	0.008	0.03*	0.214	Moderate
TSB 9:14-d SMA CH28-d	− 0.013 [− 0.023, − 0.004]	0.005	0.01*	0.313	Moderate
%INJ	0.15 [− 1.583, 1.886]	0.885	0.87	0.011	Trivial
Basketball (*n* = 13, performance measures = 171)					
(Intercept)	2.075 [1.077, 3.073]	0.509	< 0.001***		
Chronic 21-d SMA	− 0.001 [− 0.002, 0.001]	0.001	0.23	0.006	Trivial
TSB 9:21-d SMA	− 0.002 [− 0.004, − 0.000]	0.001	0.04*	0.023	Small
TSB 9:21-d SMA CH21-d	0.003 [0.002, 0.004]	0.001	< 0.001***	0.192	Moderate
%INJ	− 0.238 [− 0.611, 0.135]	0.190	0.21	0.008	Trivial

*TL* training load, *TSB* training-stress balance, *CH* change in TL measure prior to competition, *d* days, *SMA* simple moving averages, *EWMA-W* exponentially weighted moving average as per Williams et al. [23], *EWMA-L* exponentially weighted moving average as per Lazarus et al. [24]

^*^p < 0.05; ***p < 0.001; *f*^2^, Cohen’s marginal effect size

Despite these limitations, the variables which had the largest effect size in the above models appeared to be TSB (*p* = 0.03, *f*^2^ = 0.214) and the change in TSB (*p* = 0.03, *f*^2^ = 0.313) for the track and field dataset and TSB (*p* = 0.04, *f*^2^ = 0.023) and the change in TSB (*p* < 0.001, *f*^2^ = 0.192) for the basketball dataset. The differences between these model results may be related to many factors including the idiosyncrasies of the sport (e.g., competition schedules) and the sports coach and their training philosophies/structures. For example, the results of a model examining TL and performance may be quite different for a coach who favours shorter, more intense training sessions compared to a coach who prefers longer, less intense training sessions. As another example, model outputs may also be different for a coach who predominately uses three-week mesocycles compared to a coach who uses four-week mesocycles. As such, practitioners and researchers are encouraged to always be aware of the context of the sport, coach and individual athlete when interpreting any TL relationships with performance.

If modeling is used to investigate links between TL and performance, the model’s design and results should be considered in light of the theory that underpins TL research and the practicalities of applying the model results [39]. When deciding on appropriate model design, alongside accounting for a saturation effect of TL on performance [20], we recommend that the magnitude of good or poor performances should be incorporated into model designs. For instance, a linear model may be preferred over a generalized model with a binomial outcome (e.g., just “good” or “bad” performances). For example, identifying the TL that contributes to making an Olympic final (i.e., a “good” performance) versus the TL that contributes to a performance that wins an Olympic gold medal would seem more worthwhile to identify than the odds ratio for any performance being classified as being “good.” This consideration may also be a meaningful consideration when examining the relationship between injury and TL (e.g., “more” or “less” serious injuries versus only injured or not injured). When examining model outputs, both practitioners and researchers should be wary of overfitting the model test data and as mentioned previously, should aim to ensure model outputs remain both conceptually and practically valid. To highlight this concern using an example from a recent study [40], the acute (i.e., “fatigue”) and chronic (i.e., “fitness”) period lengths for an individual swimmer competing in both 50 m and 100 m sprint events were 7.7 ± 1.2 days and 73.7 ± 1.2 days for the 50 m and 5.1 ± 1.5 days and 8.7 ± 1.1 days for the 100 m. These model results seem to indicate that the *same* individual swimmer had a difference in their optimized fitness (chronic) period of ~ 65 days depending on whether they swam 50 m or 100 m in competition. This would seem an unlikely scenario and impractical for coaches to use to design training programs to improve sporting performance. In situations like this, it may be worth placing constraints on any models (e.g., a certain range of acute or chronic period options) so that model outputs remain conceptually valid and practical for end-users, i.e., sports coaches and athletes.

## Where Does Subjective Training Load Monitoring Fit in an Overall Decision-Making Framework?

**Fig. 1:**
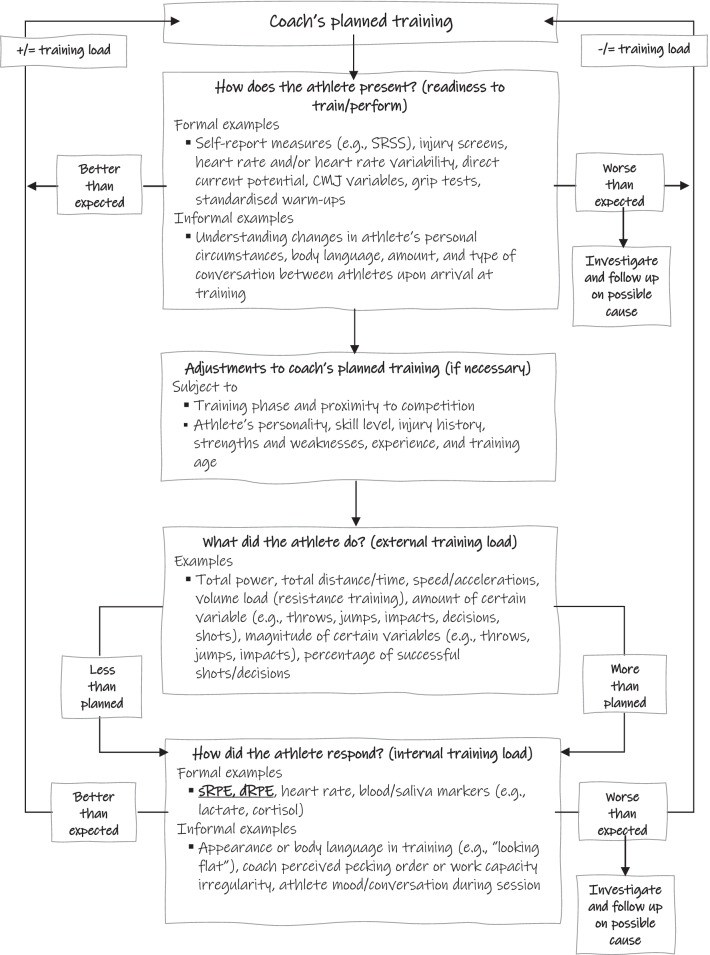
A decision-making structure for practitioners to monitor and adjust an athlete’s training. Subjective training load measures have been bolded and underlined in the figure to give context of their role in an overall decision-making process. *SRSS* short recovery and stress scale, *CMJ* countermovement jump, *sRPE* sessional ratings of perceived exertion, *dRPE* differential ratings of perceived exertion, *VAS* visual analogue scales

One of the key considerations when applying subjective TL monitoring in practice is that it is predominately a ‘*chronic’* decision-making tool (e.g., how to structure training from week-to-week or month-to-month), as described in our previous publication [12], and it relies upon post training analysis. This may become problematic when ‘*acute’* decisions are required (e.g., a coach asking “*do I need to make a change to training today? And if so, by how much?*”) or are more highly valued by high-level coaches where elite athletes’ needs may change daily [12]. Although we have previously provided examples of possible *‘acute’* and *‘chronic’* decision making tools for different types of sports [12], there is still a need to understand where subjective TL monitoring is contextualized into an overall decision-making framework. Ideally, decisions about an athlete’s training, recovery and nutrition are based on three key questions: “*how does the athlete present?*”*,* “*what did the athlete do?*” and “*how did the athlete respond?*” [41]. A possible framework that answers these questions, along with the role of subjective TL, is presented in Fig. 1. Practitioners should be cognizant that any application of decision-making frameworks like these should be with the aim of helping to inform or complement coaching, rather than dictate it [41]. Further, as TL monitoring has normally been associated with “pulling athletes back,” practitioners should be able to use the relationship between internal and external measures along with readiness to train/perform measures to increase an athlete’s TL confidently and effectively, rather than applying these measures only to reduce it.

With any of the example measures in Fig. 1, regardless of if they are subjective or not, we suggest practitioners choose which to apply by asking themselves: “*if I had to bet on it with my own money, which measure would I use?*”*.* Using this pragmatic mindset, the following factors should be considered: (1) if there is an identified need for the measure, (2) if there is commitment from the coaching team to use the data for training planning and modifications, (3) if there is or will be adequate buy-in from the athletes, and (4) provided the measures are valid and reliable, the feasibility, frequency of collection and length of the measures used [11]. For example, the commitment from an experienced expert coach to use sRPE compared to other TL measures may be dependent on the number of athletes in their training squad and only prioritized if the coach has a large squad of athletes (e.g., 5 or more) and is having trouble getting a “feel” for each athlete’s response to every training session. Although a lack of formal feedback tools like sRPE may lead to training errors by coaches due to cognitive biases [42], there is also a potential threat to the development of a coach’s learned intuition by (over-) using monitoring measures [43], similar to a coach always using video to analyse an athlete’s form and potentially negatively impacting their “coach’s eye.”

## Conclusions: Practical Strategies and Future Directions

There have been several suggestions and examples presented in this article that practitioners should consider when implementing a TL monitoring program. There are also some additional practical recommendations we advocate when using subjective measures to monitor TL. The first is to make sure the measurement device (i.e., the subjective scale) is valid and it is applied as it is intended. For example, ensuring the use of the validated nonlinear category ratio (CR-10/100) scale when collecting sRPE with athletes using the verbal anchors to obtain the numerical rating, as well as providing that rating privately to prevent any possible peer influence [4, 12, 44]. Due to issues with athletes and coaches understanding a nonlinear CR-10/100 scale, numerically blinded scales may also be a good option in practice and in research [45]. Making sure athletes are aware of the correct definition for rating effort or exertion (i.e., “the conscious sensation of how hard, heavy and strenuous a physical task is” [46, 47]) and not any other sensations (e.g., pain, discomfort, force) is also essential with any use of sRPE or dRPE in applied and research settings. This would seem especially pertinent if practitioners or researchers are wishing to apply dRPE, where rating local exertion (e.g., leg RPE) may be easily confused with local pain, discomfort, or fatigue.

Education programs focused on the correct definition for rating effort, how to correctly use a subjective scale like CR-10 or CR-100 and perhaps most importantly, how subjective TL monitoring can be used to help improve performance outcomes for athletes and coaches with practical examples, should also be implemented [41]. These programs are recommended to include training tools, like Borg’s blackness test, where individuals rate and are tested on different shades of the colour black to correspond with the verbal anchors of the sRPE scale [48], for both athletes and coaches to improve their ability and consistency in the subjective measure. If all these recommendations can be applied, the use of subjective TL monitoring could also be extended beyond just the monitoring and manipulation of training (e.g., considering the conceptual basis for sRPE is derived from agreement with heart rate [4] and the greater the exercise intensity, the greater the rate at which muscle glycogen is depleted [49], sRPE may help inform nutritional strategies peri-training with higher sRPE scores meaning an athlete may require more carbohydrate post training). Lastly, we reiterate that any TL monitoring should be based on valid measures (i.e., if using a subjective measure, ensure it has had its psychometric properties assessed) along with being implemented in practice with the aim of informing coaching decisions, and not dictating them [41].

With regard to future research directions, we suggest a conceptual model for subjective TL monitoring needs to be validated against performance; especially as it has been suggested that internal, rather than external, TL ultimately determines the functional outcome of training [1]. We do recognize that a practical validation from common athlete preparation for competition already exists (e.g., tapering before a competition to reduce fatigue is a longstanding practice in elite sport) [50] and that the relationship between internal and external TL may be more meaningful to performance than either construct in isolation [19]. As such, we revisit our previous suggestion for practitioners and researchers to compare the relationship between internal and external TL with training impulse, rather than singular volume/duration or intensity variables, especially regarding external measures. We also suggest considering the differences between internal and external TL reductions during a taper. For instance, external TL reductions common to tapers should naturally increase an athlete’s internal TSB and potentially their performance. However, this increase in internal TSB will be a function of the athlete’s perception of training when using subjective measures. As sRPE may also theoretically be influenced by any psychosocial stress an athlete is under (e.g., media commitments, nerves before a major competition), this may mean external TL may need to be modified even further by coaches, based on internal responses, to get an athlete in an optimal pre-competition state. As competition becomes more imminent, reducing cognitive work (e.g., less technique modifications or video analysis of technique) and modifying coaching feedback (e.g., more frequent positive reinforcement and strategic use of objective performance measures to boost athlete confidence) may be possible methods to augment any external TL reductions in a taper to increase internal TSB [50, 51]. For practitioners and researchers interested in monitoring an athlete’s technical or cognitive TL during tapers, dRPE may be a worthwhile tool to examine and employ in these situations.

Although we have made several recommendations in these areas, the most appropriate smoothing methods, measures, and models all require further exploration to determine how these methods align with a variety of sports. With regard to the smoothing methods, both the robust exponential decreasing index [52] and Kaufman’s adaptive moving average (KAMA) [53] warrant future investigation. One feature of KAMA that is of particular interest is it accounts for the volatility, or the standard deviation, of TL values in the smoothing period. This is important considering that an undulation in TL is conceptually desirable in performance periodization [54] and the link between strain (which is a product of TL standard deviation) and injury/illness outcomes [55, 56]. Regarding the measures of TL, both TSB and differential load appear adequate measures of change in TL but, due to their nature, may not be easily interpreted by, or intuitive for, coaches and athletes (e.g., a TSB score of + 50 may indicate quite different TL situations for different athletes in the same training squad). To account for the sometimes large fluctuations in these measures with daily calculations, another potential option that is worthy of consideration, at least with the TSB, is smoothing the measure over a period (e.g., 7-days), which is a similar concept to the moving average convergence divergence (MACD) [57] tool from financial markets. Further and as mentioned in our previous publication [12], separating technical (e.g., sports practice) and non-technical training (e.g., strength and power training, hypertrophy training, non-technical/games-based conditioning, recovery) TL and differentiating between them in performance or injury models should be considered.

Lastly, regarding the potential future modeling approaches for TL data, we suggest practitioners and researchers familiarize themselves with time series models and their use in other industries (e.g., financial markets). Although most time series modeling is concerned with estimating future outcomes of the same time series (e.g., the future price of the same financial stock), dynamic casual effect methods [58], which consider multiple concurrent times series (e.g., TL and performance) and the (lagged) effect of one time series on another, is a statistical approach that would seem to be worthy of exploration. We also suggest that researchers should make a concerted effort to examine the efficacy of different smoothing methods, measures of internal subjective intensity of TL (e.g., dRPE), measures of change in TL (e.g., TSB, differential load or MACD) and different models. Further, using standardized research methods with sample datasets and providing open-source code with any research outputs will enhance knowledge of the effects of TL on sporting performance.

## Abbreviations

TL: Training load
TSB: Training stress balance
TL-I: Training load–injury relationship
sRPE: Sessional ratings of perceived exertion
dRPE: Differential ratings of perceived exertion
AICc: Akaike Information Criterion
SMA: Simple moving average
EWMA: Exponentially weighted moving averages
EWMA-W: Exponentially weighted moving averages as per Williams
EWMA-L: Exponentially weighted moving averages as per Lazarus
ACWR: Acute to chronic workload
TE_*I*_: Training efficiency index
CR10: 10-Point category ratio scale
CR100: 100-Point category ratio scale
KAMA: Kaufman’s adaptive moving average
MACD: Moving average convergence divergence
